# 2014 Future Earth Young Scientists Conference on Integrated Science and Knowledge Co-Production for Ecosystems and Human Well-Being †

**DOI:** 10.3390/ijerph111111553

**Published:** 2014-11-10

**Authors:** Ivy Shiue, Leah Samberg, Benard Kulohoma, Diana Dogaru, Carina Wyborn, Perrine Hamel, Peter Søgaard Jørgensen, Paul Lussier, Bharath Sundaram, Michelle Lim, Antonio Tironi

**Affiliations:** 1 School of Energy, Geoscience, Infrastructure and Society, Heriot-Watt University, Riccarton, Edinburgh EH14 4AS, Scotland, UK; 2 College of Forestry and Conservation, University of Montana, Missoula, MT 59812, USA; E-Mails: lsamberg@gmail.com (L.S.); cwyborn@wwfint.org (C.W.); 3 International Centre of Insect Physiology and Ecology, Nairobi, Kenya; E-Mail: bkulohoma@iscb.org; 4 Centre for Biotechnology and Bioinformatics, University of Nairobi, Nairobi, Kenya; 5 Environment & GIS Department, Institute of Geography, Romanian Academy, Bucharest 023993, Romania; E-Mail: dianadogaru77@yahoo.com; 6 The Natural Capital Project, Woods Institute for the Environment, Stanford University, Stanford, CA 94305, USA; E-Mail: perrine.hamel@stanford.edu; 7 Center for Macroecology, Evolution and Climate, Department of Biology, University of Copenhagen, 2100 Copenhagen, Denmark; E-Mail: psjorgensen@bio.ku.dk; 8 Yale Climate & Energy Institute, Yale University, New Haven, CT 06520, USA; E-Mail: paul.lussier@yale.edu; 9 Azim Premji University, Bangalore 560100, India; E-Mail: b.sundaram@apu.edu.in; 10 Centre for Water Law, Policy and Science, University of Dundee, Dundee DD1 4HN, UK; E-Mail: m.lim@dundee.ac.uk; 11 Fundación CTF, Padre Mariano 391 #704, Providencia, Santiago 3425, Chile; E-Mail: atironi@ctf.cl

**Keywords:** future earth, policy, integrated science, ecosystem, well-being, health, green economy, anthropocene

## Abstract

Effective integration in science and knowledge co-production is a challenge that crosses research boundaries, climate regions, languages and cultures. Early career scientists are crucial in the identification of, and engagement with, obstacles and opportunities in the development of innovative solutions to complex and interconnected problems. On 25–31 May 2014, International Council for Science and International Social Science Council, in collaboration with the International Network of Next-Generation Ecologists and Institute for New Economic Thinking: Young Scholars Initiative, assembled a group of early career researchers with diverse backgrounds and research perspectives to reflect on and debate relevant issues around ecosystems and human wellbeing in the transition towards green economy, funded by the German Research Foundation, at Villa Vigoni, Italy. As a group of young scientists, we have come to a consensus that collaboration and communication among a diverse group of peers from different geographic regions could break down the barriers to multi-disciplinary research designed to solve complex global-scale problems. We also propose to establish a global systematic thinking to monitor global socio-ecological systems and to develop criteria for a “good” anthropocene. Finally, we aim to bridge gaps among research, the media, and education from a governance perspective linking with “sustainable development goals”.

## 1. Introduction

On 25–31 May, 2014, the 2nd DFG/ICSU/ISSC Young Scientists Networking Conference on Integrated Science, on the topic of “Ecosystems and human wellbeing in the transition towards green economy” was held at Villa Vigoni, Italy (http://www.icsu.org/news-centre/news/top-news/call-for-applications-young-scientists-networking-conference). We reiterated that integration in science and knowledge co-production for the global earthy system needs to start now and among early career researchers, even though it would seem to cast a huge challenge cross research boundaries, climate regions, cultures, time and space. In particular, potential obstacles and opportunities would need to be identified as early as possible in order to allow for solution-oriented mind-sets to develop. For example, the different languages, cultures, education and research trainings, work processes, personalities, collaboration politics, grant schemes, and so on, perceived as barriers, could potentially block early career researchers from developing skills necessary to make changes in the world. Conversely, opportunities to participate in knowledge co-production could foster skills necessary to develop novel solutions to solve problems from the existing traditional disciplines and harmonising conflicts due to misunderstandings [1].

The International Council for Science (ICSU; http://www.icsu.org/) and the International Social Science Council (ISSC; http://www.worldsocialscience.org/), in collaboration with the International Network of Next Generation Ecologists (INNGE; http://innge.net/) and the Institute for New Economic Thinking: Young Scholars Initiative (INET YSI; http://ineteconomics.org/ysi), assembled a group of early career researchers with diverse backgrounds and research perspectives to reflect on ecosystems and human wellbeing in the transition towards green economy and to debate relevant issues on Integrated Science that are funded by the German Research Foundation. This year, in 2014, the conference themes have included questioning key assumptions, theories, and models underlying the current research on ecosystems, human wellbeing and the transformation towards green economies; dynamics of governance, justice, governance at global and local levels, and the development of research methodologies to assess global changes and to transform the earthy system towards sustainability.

## 2. Social-Ecological Systems and the Anthropocene

The socioal-ecological perspective describes the way in which social and natural habitats shape human behaviors, and are in turn shaped by those behaviours, in particular at the system-level [2]. During the week-long conference, we discussed the application of this type of systems-thinking to re-identify existing global problems and obstacles in connecting science and research with public policy and human behaviour [3]. We also assessed socioal-ecological causal chains, stretching from the system level to human activities and lifestyle behaviours, and further to influence epigenetic phenomena and the adaption to the surrounding environments that would lead to variations in human health [4,5,6]. In this human era, which we call the Anthropocene, the human impact on natural processes is only accelerating [7,8]. The drivers of global climate change are highly correlated with economic output, and the risks from climate change to health and survival in populations are diverse and inequitable, as are the social and political ramifications [9,10]. The complex process of integrating governance, authority structures, social values (e.g., justice, equality, *etc.*) and education in the attempt to build a “good Anthropocene” requires the examination of diverse criteria across all of Earth’s systems, and an evidence-based approach to integrating information across disciplines.

## 3. Multi-Disciplinary Capacity Building

Knowledge generation and sharing rely on both rigorous research and two-way education between academics and policy-makers. This requires clear definitions and common goals towards human and environmental development. Integrated science is not a new concept. It has started in the 1950s and mainly from the health science field when integrated patient care was much needed [11]. Later in the 2000s, evidence-based practices and decision-making process by integrated knowledge have been provided in order to aid health and nursing progression [12]. This has radically changed from traditional training to modern methods for medical, health, nursing staff and even trainees. Today, this concept has been embedded into natural and social sciences when the common goal is to manage different types of environments, such as biodiversity conservation, farming, and so forth [13,14]. The additional challenge in education is how integrated science education could improve scientific literacy via the pathway of educating future teachers to be aware of the current debate and to form the most suitable opinion for the next generation [15].

## 4. Vision for the Immediate Next Step

We have come to an era where knowledge from traditional disciplines is not sufficient to solve real-life problems [16]. The future is unpredictable and several steps in learning managing Earth’s systems would need to be carefully thought through. In this context, therefore, we have proposed that our first goal would be to recognise the importance and necessity of conducting integrated science and knowledge co-production with the same language among early career researchers and to identify and then eliminate any potential barrier or threat that would delay using multi-disciplinary research to solve real-life global problems under the theme of ecosystems and human well-being. We have now also organised several research groups to look into climate change adaptation, ecosystem services and poverty alleviation, interventions, indicators, leverage points for global sustainability, meat production, uncertainty in knowledge co-production, tipping points for resilience, and so on, at the global scale (more information via: http://innge.net/wiki/index.php/Green_Economy).

Moreover, we aim to reach out to the general public to rectify a good anthropocene with the agreed on including and excluding criteria to be implemented in both research and human life experience that would help us and the next generation live through to the next century. Meanwhile, we would like to explore how developing a solution-oriented mind-set with multi-disciplinary ability could be cultivated in higher education through appropriate teaching. Finally, but not the least, we would expect to collaborate between multi-disciplinary researchers and multi-disciplinary journalists to prevent reporting bias. For this, we have been developing a global platform—Future Earth Emergence Lab (FEEL)—which would aim to disseminate appropriate sustainability research information. FEEL could serve the purpose of mediating between science and media narratives toward wider and deeper public uptake, while also hosting scientific dialogue, debates, webinar events, and hosting cross-collaboration networks towards making climate science information more social and media distribution-friendly. The FEEL platform would also comprise Future Earth (http://www.futureearth.org/) editorial messaging in the form of text, internally- and externally-produced media that supports sustainability, upload of video reports on global change and sustainability programs and the people who create them around the world, invited scientific experts, journalists and other non-scientist personalities expressing ideas related to sustainability solutions. These above-mentioned visionary actions would be great and continuous challenges in the coming decades, and we would urge supports from all aspects to join.
