# Addendum: Shiue, I.; *et al.* 2014 Future Earth Young Scientists Conference on Integrated Science and Knowledge Co-Production for Ecosystems and Human Well-Being. *Int. J. Environ. Res. Public Health* 2014, *11*, 11553–11558

**DOI:** 10.3390/ijerph120202088

**Published:** 2015-02-12

**Authors:** Ivy Shiue, Leah Samberg, Benard Kulohoma, Diana Dogaru, Carina Wyborn, Perrine Hamel, Peter Søgaard Jørgensen, Paul Lussier, Bharath Sundaram, Michelle Lim, Antonio Tironi

**Affiliations:** 1 School of Energy, Geoscience, Infrastructure and Society, Heriot-Watt University, Riccarton, Edinburgh EH14 4AS, Scotland, UK; 2 College of Forestry and Conservation, University of Montana, Missoula, MT 59812, USA; E-Mails: lsamberg@gmail.com (L.S.); cwyborn@wwfint.org (C.W.); 3 International Centre of Insect Physiology and Ecology, Nairobi, Kenya; E-Mail: bkulohoma@iscb.org; 4 Centre for Biotechnology and Bioinformatics, University of Nairobi, Nairobi, Kenya; 5 Environment & GIS Department, Institute of Geography, Romanian Academy, Bucharest 023993, Romania; E-Mail: dianadogaru77@yahoo.com; 6 The Natural Capital Project, Woods Institute for the Environment, Stanford University, Stanford, CA 94305, USA; E-Mail: perrine.hamel@stanford.edu; 7 Center for Macroecology, Evolution and Climate, Department of Biology, University of Copenhagen, Copenhagen 2100, Denmark; E-Mail: psjorgensen@bio.ku.dk; 8 International Network of Next-Generation Ecologists, Universitetsparken 15, Building 3, Copenhagen 2100, Denmark; 9 Yale Climate & Energy Institute, Yale University, New Haven, CT 06520, USA; E-Mail: paul.lussier@yale.edu; 10 Azim Premji University, Bangalore 560100, India; E-Mail: b.sundaram@apu.edu.in; 11 Centre for Water Law, Policy and Science, University of Dundee, Dundee DD1 4HN, UK; E-Mail: m.lim@dundee.ac.uk; 12 Fundación CTF, Padre Mariano 391 #704, Providencia, Santiago, Chile; E-Mail: atironi@ctf.cl

The authors would like to add the following affiliation for Peter Søgaard Jørgensen of paper [1]:

^8^ International Network of Next-Generation Ecologists, Universitetsparken 15, Building 3, Copenhagen 2100, Denmark

The authors apologize for any inconvenience caused to the readers.
